# Clinical characteristics and long-term disabilities in children following *Bothrops atrox* envenomation in Manaus, western Brazilian Amazon

**DOI:** 10.1590/1678-9199-JVATITD-2025-0092

**Published:** 2026-07-17

**Authors:** Débora Nery Oliveira, Jady Shayenne Mota Cordeiro, Andrea Gabriela Mota Saraiva, Hiran Sátiro Souza da Gama, Daniel Barros Castro, Alexandre Vilhena Silva-Neto, André Sachett, Marco Aurélio Satim, Felipe Murta, Wuelton Marcelo Monteiro, Jacqueline de Almeida Gonçalves Sachett

**Affiliations:** 1 School of Health Sciences, State University of Amazonas, Manaus, AM, Brazil.; 2 Department of Teaching and Research, Dr. Heitor Vieira Dourado Tropical Medicine Foundation, Manaus, AM, Brazil.; 3 Nilton Lins University (UNL), Manaus, AM, Brazil.; 4 Coordination of Society, Environment and Health, National Institute for Amazonian Research, Manaus, AM, Brazil.; 5 Graduate Program in Basic and Applied Immunology, Federal University of Amazonas, Manaus, AM, Brazil.; 6 Department of Pharmaceutical Sciences, Federal University of Amazonas, Manaus, AM, Brazil.; 7 Duke Global Health Institute, Duke University, Durham, United States.; 8 Department of Teaching and Research, Alfredo da Matta Hospital Foundation, Manaus, AM, Brazil.

**Keywords:** Snakebite, Children, Long-term disabilities, Neglected tropical diseases, Bothrops atrox

## Abstract

**Background:**

In the Brazilian Amazon, most snakebites are caused by *Bothrops atrox*. Although pediatric cases are less frequent, children are more vulnerable to severe complications and long-term disabilities. This study aims to describe the clinical profile of *B. atrox* envenomation in children treated at a tertiary hospital in Manaus, in Western Brazilian Amazon, and to characterize the resulting long-term musculoskeletal impairments in a subgroup of these patients.

**Methods:**

We retrospectively analyzed sociodemographic and clinical data from patients up to 12 years and 11 months of age treated between January 2010 and December 2023. A total of 258 children who were victims of *B. atrox* envenoming were eligible; however, a subgroup of 27 children underwent in-person musculoskeletal evaluations starting three months after hospital discharge.

**Results:**

In the subgroup of children evaluated, the majority were male (63%), were aged over ten years (59.3%), and were from rural areas (96.3%). Over half (51.8%) received medical care within six hours after the bite. The lower limbs were most frequently affected (96.3%). Common local symptoms included pain (100%), edema (96.3%), bleeding (37%), and bruising (29.6%). Secondary infections occurred in 18.5% of cases. Most envenomations were classified as moderate in severity (44.4%). In this subgroup, long-term disabilities were identified in 21 children (77.7%), who presented primarily with intermittent chronic pain (55.5%). Physical examination revealed scars (59.3%), edema (22.2%), and deformities (3.7%). Sensory alterations were noted in tactile (11.1%), pain (25.9%), thermal (22.2%), and vibratory (29.6%) sensitivity. Range of motion was impaired in 37% of cases, and one child exhibited abnormal posture and reflexes.

**Conclusions:**

This study highlights a broad spectrum of persistent musculoskeletal sequelae following *Bothrops* envenomation in children. Our findings underscore the urgent need for comprehensive care, follow-up, and rehabilitation programs for pediatric snakebite victims in the Amazon region.

## Background

Snakebite envenoming affects over five million people globally each year, disproportionately impacting young individuals of working age [1]. While snakebites in children are relatively underreported, they represent a significant public health concern. In sub-Saharan Africa, approximately 30.4% of snakebite victims are children [2] and in Brazil, pediatric cases account for 15-30% of total envenomations [3-5]. The Brazilian Amazon reports the highest incidence of snakebites in the country, with *Bothrops atrox*, commonly known as *jararaca*, responsible for approximately 90% of cases [3, 6-8]*.* In 2020, the state of Amazonas recorded an incidence rate of 13.5 cases per 100,000 inhabitants per year among children aged 14 years or younger [3]. 

Although snakebites are less frequent in the pediatric population, they tend to result in more severe clinical outcomes due to the smaller body mass and higher venom-to-weight ratio in children. This vulnerability increases the risk of complications and long-term disabilities [5, 9, 10]. Even with timely antivenom administration, *Bothrops* envenomations in children can lead to tissue necrosis, compartment syndrome, hemodynamic instability, pulmonary edema, and acute kidney injury [1-13]. Children under 12 years of age are 2.24 times more likely to require amputation following a snakebite than adults [14,15].

Timely access to medical care is crucial for preventing complications; however, the vast geography of the Amazon region poses significant barriers. Snakebite envenomations frequently occur in remote areas with limited infrastructure and long distances to health centers equipped for antivenom administration [3,16,17]. The shortage of pediatric specialists and intensive care resources further exacerbates the challenge of providing adequate care in this region [5]. 

Snakebites can lead to long-term disabilities that affect physical, motor, and psychological functions, potentially impairing growth and development into adulthood [5,13,18-21]. Previous studies have documented secondary infections, compartment syndrome, hypertrophic scarring, skin grafting, and limb dysfunction in pediatric snakebite survivors [11, 21, 22]. Long-term disabilities in children may include peroneal nerve palsy, flaccid hemiplegia, extensive tissue loss, motor deficits, and even limb amputation [23-25]. This study aims to describe the clinical profile of *Bothrops atrox* envenomation in children treated at a tertiary hospital in Manaus, in the western Brazilian Amazon, and to characterize the resulting long-term musculoskeletal disabilities in a subset of these patients.

## Methods

### Study design and location

This descriptive study was conducted at the Dr. Heitor Vieira Dourado Tropical Medicine Foundation (FMT-HVD), a reference center for the treatment of venomous animal envenomations in the state of Amazonas. The institution is located in Manaus, the state capital, in the Western Brazilian Amazon.

### Population and sample

The study included 258 children who experienced *B. atrox* envenomation up to 12 years and 11 months of age [26] and were hospitalized at FMT-HVD between January 2010 and December 2023. In a second phase, efforts were made to contact patients with valid phone numbers recorded in their medical records to invite them to participate in a post-discharge physical assessment. 

### Eligibility criteria

To be eligible, children must have sustained a *B. atrox* snakebite before turning 13 years old. For inclusion in the musculoskeletal assessment, participants (and their legal guardians) had to provide consent and have no history of unrelated injuries to the affected limb after hospital discharge. Exclusion criteria included neurological conditions that could interfere with motor assessments, inability to attend the evaluation and other comorbidities such as diabetes or diseases affecting the peripheral vascular system. Indigenous patients were also excluded due to the difficulty of accessing indigenous areas, as well as identifying their places of residence for in-person visits; furthermore, there is an ethical limitation in accessing these areas.

### Data collection

Data were collected in two phases:

Phase 1: Sociodemographic and clinical data were extracted from 258 electronic medical records (iDoctor system), including: demographic information, snakebite circumstances, time to medical care, anatomical site of the bite, local and systemic manifestations, complications, antivenom administration, envenoming classification (mild 2-4 vials, moderate 4-8 vials and severe 12 vials) [27], and outcomes.

Phase 2: Telephone contact was attempted using numbers from the medical records. Musculoskeletal assessments were conducted in person - either at the participant's residence or at FMT/HVD - by the principal investigator. Evaluations lasted approximately 30-40 minutes and included participants from Manaus and surrounding municipalities (Iranduba, Itacoatiara, Presidente Figueiredo, Careiro da Várzea, Rio Preto da Eva, and Manacapuru).

### Musculoskeletal functionality evaluation

A structured assessment form was used, including anamnesis and a physical examination of the musculoskeletal system. The evaluation covered medical history, pain, edema, deformities, range of motion (ROM), muscle strength, sensory function, and neuromuscular reflexes.

Pain intensity was assessed using a pediatric visual analog scale with six facial expressions, classified as: 0 (absent), 1-3 (mild), 4-7 (moderate), and 8-10 (severe). Pain was further categorized as intermittent (occurring at specific times of day) or continuous (persistent) [28, 29]. Edema was assessed through inspection, palpation, and bilateral measurement using a tape measure, comparing the affected and contralateral limbs. A difference in circumference indicated the presence of edema [30]. ROM was measured with a goniometer and considered altered if a ≥ 15% difference was observed between the limbs [31, 32]. Muscle strength was assessed using the Kendall method and rated according to the Medical Research Council (MRC) scale (0 to 5). Scores below 4 were considered abnormal [32-34]. Neuromuscular reflexes were evaluated with a reflex hammer and classified as normal, altered, or absent depending on the response [29].

### Superficial and deep sensitivity test

*Tactile sensitivity*

Sensitivity was assessed with an esthesiometer and classified as: Grade 0: sensitivity to 0.05-2.0 g monofilaments; Grade 1: sensitivity to 4.0-300 g monofilaments and Grade 2: Grade 1 plus visible disability related to the snakebite. 

*Thermal sensitivity*

Sensitivity was assessed using test tubes with water at 5-10 °C (cold) and 40-42.5 °C (hot). Results were classified as normal or absent depending on the patient’s ability to correctly identify the temperature. 

*Pain sensitivity*

Sensitivity was tested with a no. 11 crochet needle. Pain perception was recorded as present if the patient recognized the painful stimulus, and absent otherwise [35]. 

*Vibratory sensitivity*

Sensitivity was assessed using a 256 Hz tuning fork applied to bony prominences. Patients were instructed to report when the vibration ceased. Outcomes were classified as normal, anesthesia (absence), hypoesthesia (early loss), or hyperesthesia (prolonged perception). 

*Kinetic-postural sensitivity*

Sensitivity was assessed by passively moving the affected limb and asking the patient to identify the limb’s position. Results were categorized as normal or absent [29]. 

### Outcomes

Long-term disability can be defined as the presence of chronic clinical manifestations, characterized by conditions that persist or manifest for more than six weeks after envenomation and that require ongoing monitoring and care after hospital discharge [19-21].

Individuals were considered to have musculoskeletal disorders if they presented with abnormalities in the musculoskeletal assessment, including complaints of intermittent or persistent pain, the presence of edema, physical deformities, and impairment of range of motion (ROM), muscle strength, sensory function, and neuromuscular reflexes.

### Data Analysis

Data were entered into REDCap (Vanderbilt University, USA) and analyzed using R (version 4.2) and RStudio (version 2023.3). Comparisons between groups with and without long-term disabilities were performed using chi-square (χ²) or Fisher’s exact tests. A p-value < 0.05 was considered statistically significant.

## Results 

During the study period, 269 medical records were reviewed. Of these, 11 records were not included due to a lack of information or because the causative agent was not of the *Bothrops* genus. The analysis was performed on the remaining 258 medical records, from which sociodemographic and clinical data were extracted. Of these, 215 patients had a registered telephone contact, but 72 were excluded: 34 because they were indigenous and 38 for residing in municipalities far from the capital. Thus, 143 patients were successfully contacted. Of these, only 29 underwent musculoskeletal evaluation, with two children being excluded: one due to a fracture in the affected limb and another for being too young (four years old) to complete the responses.

### General profile of injured children

Most snakebites involved male patients (63.2%), with the most affected age group being children over ten years old (60.5%). Most snakebites occurred in the interior municipalities of the state of Amazonas (71.3%), mainly in Manacapuru, Novo Airão, and Presidente Figueiredo, predominantly in rural areas (94.9%). A total of 67.4% of the patients used medications (analgesics and anti-inflammatory drugs) before receiving care at a healthcare facility. Additionally, some patients performed pre-hospital interventions, which included the use of tourniquets and the application of products to the bite site. Most victims took more than six hours to receive medical care (57.8%) (Table 1).

**Table 1.** General characteristics of snakebites in the pediatric population and in children assessed for long-term disabilities.

Variable	Total (n = 258, %)	Assessed for long-term disabilities (n = 27, %)
**Gender**		
Female	95 (36.8)	10 (37.0)
Male	163 (63.2)	17 (63.0)
**Age groups (years)**		
< 6	39 (15.1)	5 (18.5)
6-10	63 (24.4)	6 (22.2)
> 10	156 (60.5)	16 (59.3)
**City**		
Manaus	74 (28.7)	9 (33.3)
Other cites	184 (71.3)	18 (66.7)
**Area of occurrence**		
Rural	245 (94.9)	26 (96.3)
Urban	13 (5.1)	1 (3.7)
**Pre-hospital care**		
Use of medications	174 (67.4)	16 (59.3)
Tourniquet	14 (5.4)	3 (11.1)
Topical medicines	30 (11.6)	6 (22.2)
**Time until medical care (hours)**		
< 6	109 (42.2)	14 (51.9)
6-12	58 (22.5)	4 (14.8)
12-24	39 (15.1)	6 (22.2)
24-48	27 (10.5)	2 (7.4)
> 48	25 (9.7)	1 (3.7)
**Anatomical region of the bite**		
Upper limb	13 (5.0)	-
Lower limb	208 (80.6)	26 (96.3)
Other	37 (14.4)	1 (3.7)

The most common local manifestations were pain (100%), edema (97.7%), local bleeding (29.5%), and bruising (22.5%). The most frequent systemic manifestations were fever (14.2%), headache (18.2%), vomiting (17.8%), nausea (13.9%) and lymph node enlargement (7%). Regarding complications resulting from the snakebite, secondary infection was present in 22.5% of children, and 5.4% presented with compartment syndrome. Regarding the classification of the envenoming, the majority of cases were considered moderate (54.7%), with little difference between mild (24.4%) and severe (20.9%) cases. No deaths occurred (Table 2).

**Table 2.** Clinical characteristics of snakebites in pediatric population and in children assessed for long-term disabilities.

Variable	Total (n = 258, %)	Assessed for long-term disabilities (n = 27, %)
**Local manifestations**		
Pain	258 (100.0)	27 (100.0)
Edema	252 (97.7)	26 (96.3)
Local bleeding	76 (29.5)	10 (37.0)
Local serous secretion	37 (14.3)	3 (11.1)
Ecchymosis	58 (22.5)	8 (29.6)
Blisters	25 (9.7)	2 (7.4)
**Systemic manifestations**		
Fever	37 (14.3)	3 (11.1)
Headache	47 (18.2)	6 (22.2)
Vomit	46 (17.8)	6 (22.2)
Nausea	36 (13.9)	7 (25.9)
Enlarged lymph nodes	18 (7.0)	2 (7.4)
Gingival bleeding	13 (5.0)	2 (7.4)
Sweating	7 (2.7)	1 (3.7)
Diarrhea	6 (2.3)	1 (3.7)
Abdominal pain	8 (3.1)	-
Convulsion	3 (1.2)	-
Hematuria	4 (1.5)	-
Conjunctival bleeding	2 (0.8)	-
**Snakebite complications**		
Secondary infection	58 (22.5)	5 (18.5)
Compartment syndrome	14 (5.4)	1 (3.7)
Necrosis	9 (3.5)	1 (3.7)
Acute kidney injury	10 (4.4)	3 (13.6)
**Severity classification on admission**		
Mild	63 (24.4)	8 (29.6)
Moderate	141 (54.7)	12 (44.5)
Severe	54 (20.9)	7 (26.0)
**Hospital stay (days)**		
< 4	109 (42.2)	9 (33.3)
4-7	84 (32.6)	14 (51.9)
> 7	65 (25.2)	4 (14.8)

Regarding laboratory tests, leukocyte counts (71%), creatine kinase levels (51.5%), and lactate dehydrogenase levels (76.9%) were increased at the time of admission (Table 3).

**Table 3.** Laboratory characteristics of snakebites in the pediatric population and in children assessed for long-term disabilities.

Variable	Total (n = 258, %)	Assessed for long-term disabilities (n = 27, %)
**Erytdrocytes (cells/mm³)**		
Lower	47 (19.0)	1 (4.0)
Normal	200 (81.0)	24 (96.0)
**Hemoglobin (g/dL)**		
Lower	114 (45.8)	7 (26.9)
Normal	135 (54.2)	19 (73.1)
**Hematocrit (%)**		
Lower	91 (36.55)	4 (15.38)
Normal	158 (63.5)	22 (84.62)
**Leukocytes (cells/mm³)**		
Normal	72 (29.0)	12 (48.0)
Higher	176 (71.0)	13 (52.0)
**Platelets (cells/mm³)**		
Lower	15 (6.0)	4 (16.0)
Normal	233 (94.0)	21 (84.0)
**Creatine kinase (U/L)**		
Normal	66 (48.5)	13 (72.2)
Higher	70 (51.5)	5 (27.8)
**Aspartate aminotransferase (IU/L)**		
Normal	96 (63.6)	12 (70.6)
Higher	55 (36.4)	5 (29.4)
**Alanine aminotransferase (IU/L)**		
Normal	137 (94.5)	17 (100.0)
Higher	8 (5.5)	-
**Creatinine (mg/dL)**		
Normal	217 (95.6)	19 (86.4)
Higher	10 (4.4)	3 (13.6)
Urea (mg/dL)		
Normal	216 (96.0)	22 (100.0)
Higher	9 (4.0)	-
**Lactate dehydrogenase (U/L)**		
Normal	21 (23.1)	1 (9.1)
Higher	70 (76.9)	10 (90.9)
**Clotting time**		
Coagulable	115 (94.3)	7 (87.5)
Incoagulable	7 (5.7)	1 (12.5)

Reference values: erythrocytes 4.2-5.5 millions/mm³; hemoglobin 12.5-15.5 g/dl; Hematocrit 36-37%; leukocytes 4,000-10,000/mm³; platelets 150,000-450,000/mm³; creatinine kinase 24-190 U/L; aspartate aminotransferase 2-38 IU/L; alanine aminotransferase 2-44 IU/L; creatinine 0.3-1.0 mg/dL; urea 10-45 mg/dL; lactate dehydrogenase 211-223 U/L; clotting time: coagulable 5-10 minutes.

### Children with musculoskeletal assessment

The musculoskeletal assessment was conducted on 27 children and performed in person. Among these envenomations, the majority were classified as moderate (44.4%), followed by mild (29.6%) and severe (25.9%) (Table 2). According to the epidemiological data of the snakebite envenomations in the children who were assessed (n = 27), there were no significant differences compared to the overall profile of the total study population (n = 258). The majority of the envenomations occurred in males (63%). Most of the envenomations took place in the interior regions of the state of Amazonas (66.7%), in the municipalities of Manacapuru, Itacoatiara, Iranduba, and Rio Preto da Eva, originating from rural areas (96.3%). Twenty-two percent of the patients received some form of treatment before hospital care, and 59.3% of the patients took medications before entering the healthcare facility.

In the musculoskeletal evaluations, patients reported late complaints following the envenomation, such as paresthesia, difficulty walking, muscle weakness, and intermittent pain in the affected site or limb. Scars (59.3%), edema (22.2%), and deformities (3.7%) were found in the affected limbs of the children. Concerning the musculoskeletal assessment, abnormalities were found in the range of motion of the affected limb (38.5%). Only one child exhibited abnormalities in neuromuscular reflexes (Table 4). 

**Table 4.** Long-term findings of the neuromusculoskeletal assessment of the study participants (n = 27).

Variable	Frequency (n, %)
**Late complaints after envenomation**	
Paresthesia	4 (14.8)
Difficulty walking	3 (11.1)
Muscle weakness	1 (3.7)
Lack of sensitivity	1 (3.7)
Cyanosis	1 (3.7)
Dread	1 (3.7)
Headache	1 (3.7)
**Pain intensity**	
Absent	12 (44.5)
Mild	4 (14.8)
Moderate	7 (26.0)
Intense	4 (14.8)
**Pain characteristic**	
Intermittent	15 (55.6)
Presence of scar	16 (59.3)
Scar morphology		
Normotrophic	15 (55.6)
Atrophic	1 (3.7)
**Scar color**	
Normochromic	5 (18.5)
Hyperchromic	1 (3.7)
Presence of edema	6 (22.2)
Presence of deformity	1 (3.7)
**Range of joint motion**	
Normal	17 (63.0)
Changed	10 (37.0)
**Strength of the affected limb**	
Normal	27 (100.0)
Changed	-
**Deep osteotendinous reflexes**	
Absent	1 (3.7)
Normal	26 (96.3)

Regarding long-term changes in sensitivity, a reduction in tactile sensitivity was observed in 7.4% of cases with Grade 1 and in 3.7% with Grade 2. Thermal sensitivity decreased in 22.2%, pain sensitivity in 25.9% and kinetic-postural sensitivity in 3.7% of patients. Vibratory sensitivity decreased in 18.5% (hypoesthesia), increased in 7.4% (hyperesthesia) and absent in 3.7% (Table 5).

**Table 5.** Long-term sensory alterations in the affected limb of the study participants (n = 27).

Variable	Frequency (n, %)
**Tactile sensitivity**	
Normal (Grade 0)	24 (88.9)
Decreased (Grade 1)	2 (7.4)
Decreased (Grade 2)	1 (3.7)
**Thermal sensitivity**	
Normal	21 (77.8)
Decreased	6 (22.2)
**Pain sensitivity**	
Normal	20 (74.1)
Decreased	7 (25.9)
**Kinetic-postural sensitivity**	
Normal	26 (96.3)
Decreased	1 (3.7)
**Vibratory sensitivity**	
Normal	19 (70.4)
Anesthesia	1 (3.7)
Hypoesthesia	5 (18.5)
Hyperesthesia	2 (7.4)

Medication use before hospital admission is a predictor of long-term disability in children with *Bothrops* envenomation (Table 6).

**Table 6.** Predictors of long-term disabilities from *Bothrops atrox* snakebites in children.

Variable	No disability n = 6 (%)	Disability n = 21 (%)	*p*
**Gender**			0.4
Female	1 (16.7)	9 (42.9)	
Male	5 (83.3)	12 (57.1)	
**Age groups (years)**			0.5
< 6	-	5 (23.8)	
6-10	1 (16.7)	5 (23.8)	
> 10	5 (83.3)	11 (52.4)	
**City**			0.4
Manaus	3 (50.0)	15 (71.4)	
Capital	3 (50.0)	6 (28.6)	
**Area of occurrence**			> 0.9
Rural	-	1 (4.8)	
Urban	6 (100.0)	20 (95.2)	
**Pre-hospital care**			
Use of medications	1 (16.7)	15 (71.4)	0.027
Tourniquet	-	3 (14.3)	> 0.9
Topical medicines	2 (33.3)	4 (19.0)	0.6
**Time until medical care (hours)**			0.8
< 6	4 (66.7)	10 (47.7)	
6-12	-	4 (19.0)	
12-24	2 (33.3)	4 (19.0)	
24-48	-	2 (9.5)	
> 48	-	1 (4.8)	
**Anatomical region of the bite**			> 0.9
Lower limb	6 (100.0)	20 (95.2)	
Other	-	1 (4.8)	
**Local manifestations**			
Pain	6 (100.0)	21 (100.0)	> 0.9
Edema	6 (100.0)	20 (95.2)	> 0.9
Local bleeding	1 (16.7)	9 (42.9)	0.4
Local serous secretion	-	3 (14.3)	> 0.9
Ecchymosis	1 (16.7)	7 (33.3)	0.6
Blisters	-	2 (9.5)	> 0.9
**Systemic manifestations**			
Fever	-	3 (14.3)	> 0.9
Headache	1 (16.7)	5 (23.8)	> 0.9
Vomit	2 (33.3)	4 (19.0)	0.6
Nausea	2 (33.3)	5 (23.8)	0.6
Enlarged lymph nodes	-	2 (9.5)	> 0.9
Gingival bleeding	-	2 (9.5)	> 0.9
Sweating	-	1 (4.8)	> 0.9
Diarrhea	-	1 (4.8)	> 0.9
**Snakebite complications**			
Secondary infection	-	5 (23.8)	0.6
Compartmental syndrome	-	1 (4.8)	> 0.9
Necrosis	-	1 (4.8)	> 0.9
**Severity on admission**			0.6
Mild	1 (16.7)	7 (33.3)	
Moderate	4 (66.7)	8 (38.1)	
Severe	1 (16.7)	6 (28.6)	
**Hospital stay (days)**			0.6
< 4	3 (50.0)	6 (28.6)	
4-7	3 (50.0)	11 (52.4)	
> 7	-	4 (19.0)	
**Erythrocytes (cells/mm³)**			> 0.9
Lower	-	1 (5.0)	
Normal	5 (100.0)	19 (95.0)	
**Hemoglobin (g/dL)**			> 0.9
Lower	1 (20.0)	6 (28.6)	
Normal	4 (80.0)	15 (71.4)	
**Hematocrit (%)**			0.6
Lower	-	4 (19.0)	
Normal	5 (100.0)	17 (81.0)	
**Leukocytes (cells/mm³)**			0.6
Higher	2 (40.0)	11 (55.0)	
Normal	3 (60.0)	9 (45.0)	
**Platelets (cells/mm³)**			0.5
Lower	1 (25.0)	3 (14.3)	
Normal	3 (75.0)	18 (85.7)	
**Creatine kinase (U/L)**			> 0.9
Higher	1 (33.3)	4 (26.7)	
Normal	2 (66.7)	11 (73.3)	
**AST (IU/L)**			> 0.9
Higher	-	5 (33.3)	
Normal	2 (100.0)	10 (66.7)	
**ALT (IU/L)**			> 0.9
**Higher**			
Normal	2 (100.0)	15 (100.0)	
**Creatinine (mg/dL)**			0.3
Higher	1 (50.0)	2 (10.0)	
Normal	1 (50.0)	18 (90.0)	
**Urea (mg/dL)**			
Higher	-	-	
Normal	2 (100.0)	20 (100.0)	
**LDH (U/L)**			> 0.9
Higher	-	1 (9.1)	
Normal	-	10 (90.9)	
**Clotting time**			> 0.9
Coagulable	-	7 (87.5)	
Incoagulable	-	1 (12.5)	

**Figure 1.**
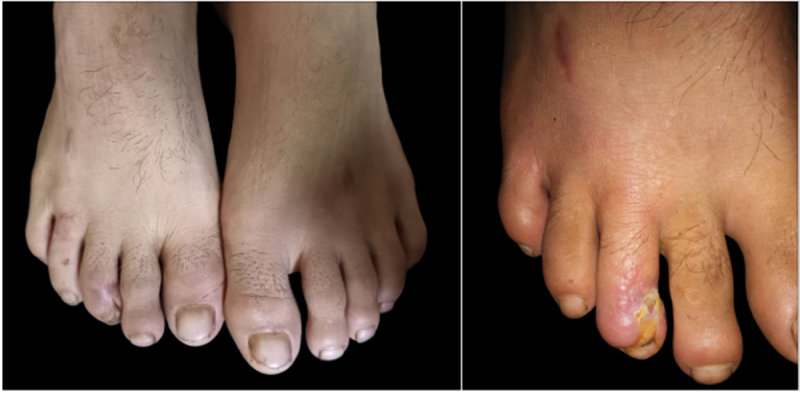
Male patient, 13 years old, with severe injury to both feet, which progressed to secondary infection during the hospital stay. The evaluation was performed eight months after the envenoming, showing chronic pain in the affected limb, deformity in the right toe, and altered and pain sensitivity.

**Figure 2.**
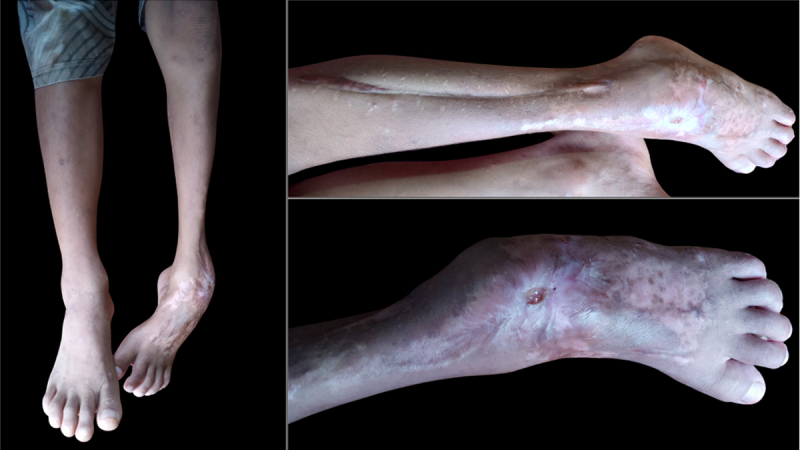
Male patient, ten years old, with a severe injury to the left leg, which progressed to compartment syndrome during the hospital stay. The evaluation was performed eight months after the envenoming, showing chronic pain in the affected limb, a fasciotomy scar, muscle atrophy, and a small open wound on the left foot, and all sensory and motor assessments were abnormal. The patient is unable to perform daily activities.

Figures 1 and 2 present two cases of long-term permanent injuries.

## Discussion

The study demonstrated that most cases involved male patients, with children over ten years of age being the most affected age group. A significant portion of the victims received medical attention within six hours of envenoming. Musculoskeletal assessments revealed delayed-onset symptoms such as paresthesia, difficulty walking, muscle weakness, and intermittent pain. Physical findings included scarring, edema, and deformities in the affected limbs. Part of the sample exhibited an impaired range of motion, while only one child presented altered neuromuscular reflexes. Long-term sensory deficits were also observed, including reduced thermal sensitivity and impaired vibratory perception.

In Brazil, snakebite envenomations have the highest incidence in the Amazon region compared to other regions of the country. Despite snakebite envenoming being a condition of compulsory notification to the Ministry of Health, there is still underreporting of cases throughout the region [3, 36]. The pediatric population is infrequently affected, with most snakebite envenomations occurring predominantly among boys living in rural areas, who are often bitten while playing, walking, or assisting their parents with agricultural labor [4, 9, 12]. 

Many envenomations in this study exhibited these characteristics, highlighting similarities with envenomations in adults [10]. It is common in the Brazilian Amazon for children to begin working early in agriculture to support their families, particularly in vulnerable populations due to basic survival needs, living in a sociocultural and economic context different from the rest of the country [37]. Consequently, children working in rural areas are more likely to encounter snakes and sustain bites.

In the rural context, a critical factor to consider is the time required to obtain medical care to prevent worsening and fatality. In Amazonas, this risk is exacerbated by the state's vast territorial extent, which complicates the transport of victims, with most arriving at the hospital more than six hours after the bite [16, 38, 39]. Another significant factor is post-envenoming practices; many individuals still use traditional medicine, apply tourniquets, perform suction, employ "milking" techniques, apply home remedies to the injury, and self-medicate to mitigate worsening [4,11,17]. These practices were evident in this study, where a considerable number of victims took more than six hours to receive initial medical attention. Although the number of children who engaged in traditional medicine or other empirical practices was low, there are still cases where caregivers employed such methods.

Snakebite envenomations primarily affect the lower limbs, and this study observed the same in children, partly because children in rural areas often play barefoot, lack boots for agricultural work, and often need to walk to school [9, 13, 22, 23]. This is similar to snakebite envenomation in adults, who also experience bites on the lower limbs, albeit for different reasons related to rural labor [10, 38]. Bucaretchi et al. [23] found that children primarily experienced pain, swelling, and bruising when bitten by *Bothrops* spp. This study observed similar findings, with pain, swelling, erythema, and warmth as local symptoms, and fever, headache, and vomiting as systemic symptoms. 

In this study, children developed complications from envenoming, with most developing secondary infections (22.5%), consistent with other studies involving children [9, 40]. Other complications, albeit less frequent, related to snakebite envenomation in children, such as compartment syndrome and acute kidney injury, were also observed. Brenes-Chacón et al. [22], showed that most children in their study developed compartment syndrome and associated snakebite complications with severity classification, i.e., moderate and severe cases tend to present complications. It is important to note that a significant portion of our population had the envenomation classified as moderate.

Studies indicate that children who experience complications from envenoming, such as secondary infections, necrosis, and compartment syndrome, are those who develop the most permanent sequelae [11, 22]. In some cases, even if amputation is not required, tissue, muscle, and nerve loss from snakebite complications may necessitate skin grafts, leading to physical impairment or loss of limb function [11, 41]. Our findings primarily reveal alterations in the range of motion of the affected limb and sensory changes (tactile, thermal, pain, and vibratory). This occurs because cellular damage following envenomation rapidly affects tissue, resulting in poor regeneration of muscle fibers and nerves, which impairs the transmission and reception of electrical impulses [42, 43]. 

Although movement limitations primarily occur during the acute phase of envenomation due to local inflammation and tissue damage, such limitations can persist for months or years post-envenoming [44, 45]. This is due to the degradation process in which muscles suffer damage, leading to alterations in muscle extensibility and elasticity. For instance, muscles in the lower limbs, originating at the knee and extending to the feet, are affected by the bite, making extension, flexion, and toe movement more challenging [46, 47]. In this study, we observed that in some cases, these alterations persist, with some victims who underwent range of motion assessments showing changes in dorsiflexion and flexion of the lower limb even years after the envenomation.

Other studies also report mobility changes that hinder daily activities for months or even years [21, 48, 49]. Additionally, other motor alterations such as chronic pain, local paralysis, and weakness at the bite site, similar to findings in this study, can be observed [20, 45, 50]. During the evaluation, reports of chronic pain during daily activities such as running, walking, and playing were noted in the affected limb. There may also be cases of chronic edema, which, alongside other symptoms, interferes with daily activities due to toxin action degrading both the vasculature and extracellular matrix (ECM), resulting in vascular and lymphatic changes leading to fluid accumulation from inflammatory processes that further exacerbate tissue damage [45, 47]. This edema persists due to difficulties in vascular regeneration.

It is evident that there is rarely a follow-up of victims after hospital discharge, which impedes the healthcare system's ability to detect deficiencies related to snakebite envenomations and their consequences. Ideally, there should be multidisciplinary follow-up post-envenoming, and physical therapy rehabilitation is an option to ensure that children grow without significant musculoskeletal damage impacting their future socioeconomic status [51]. Although there is a WHO strategy for snakebite prevention and control emphasizing victim follow-up, in the Amazon, the distance from medical centers presents a challenge for seeking appropriate treatments and therapies that can help avoid greater disability [16].

The limitations of this study are related to the difficulty of accessing all children who suffered snakebite envenomations, particularly those living in areas far from Manaus and municipalities distant from the Amazonas’ capital. Another limiting factor is the constant change in phone numbers provided upon hospital admission, which hindered in-person assessment.

## Conclusion

In this study, we found chronic pain, alterations in the range of motion, and loss of sensation in snakebite envenomations in children. Pediatric snakebite envenoming requires increased attention from healthcare services; there is a clear shortage of qualified professionals in areas with a high number of cases, which impedes access to timely treatment. This delay can result in complications and, consequently, lead to physical disabilities. Proper care during and after the acute phase of envenomation is of utmost importance. However, it is essential to have assistance mechanisms targeting the more vulnerable populations with limited access in the Amazon, ensuring they receive all necessary support for recovery to grow with minimal damage.

### Abbreviations

ALT: alanine aminotransferase; AST: aspartate aminotransferase; CK: creatine kinase; 

ECM: extracellular matrix; FMT-HVD: Dr. Heitor Vieira Dourado Tropical Medicine Foundation; LDH: lactate dehydrogenase; MRC: Medical Research Council; ROM: range of motion; WHO: World Health Organization.

## Data Availability

All data generated or analyzed during this study are included in this article.
